# Determination of optimized oxygen partial pressure to maximize the liver regenerative potential of the secretome obtained from adipose-derived stem cells

**DOI:** 10.1186/s13287-017-0635-x

**Published:** 2017-08-03

**Authors:** Sang Chul Lee, Kee-Hwan Kim, Ok-Hee Kim, Sang Kuon Lee, Ha-Eun Hong, Seong Su Won, Sang-Jin Jeon, Byung Jo Choi, Wonjun Jeong, Say-June Kim

**Affiliations:** 1 Department of Surgery, Daejeon St. Mary’s Hospital, College of Medicine, The Catholic University of Korea, Daeheung-dong 520-2, Joong-gu, Daejeon, Republic of Korea; 2 Department of Surgery, Uijeongbu St. Mary’s Hospital, College of Medicine, The Catholic University of Korea, Uijeongbu, Republic of Korea

**Keywords:** Adipose tissue-derived stem cells, Hypoxia, Liver regeneration, Sirtuin-1 (SIRT1), Secretome

## Abstract

**Background:**

A hypoxic-preconditioned secretome from stem cells reportedly promotes the functional and regenerative capacity of the liver more effectively than a control secretome. However, the optimum oxygen partial pressure (pO_2_) in the cell culture system that maximizes the therapeutic potential of the secretome has not yet been determined.

**Methods:**

We first determined the cellular alterations in adipose tissue-derived stem cells (ASCs) cultured under different pO_2_ (21%, 10%, 5%, and 1%). Subsequently, partially hepatectomized mice were injected with the secretome of ASCs cultured under different pO_2_, and then sera and liver specimens were obtained for analyses.

**Results:**

Of all AML12 cells cultured under different pO_2_, the AML12 cells cultured under 1% pO_2_ showed the highest mRNA expression of proliferation-associated markers (IL-6, HGF, and VEGF). In the cell proliferation assay, the AML12 cells cultured with the secretome of 1% pO_2_ showed the highest cell proliferation, followed by the cells cultured with the secretome of 21%, 10%, and 5% pO_2_, in that order. When injected into the partially hepatectomized mice, the 1% pO_2_ secretome most significantly increased the number of Ki67-positive cells, reduced serum levels of proinflammatory mediators (IL-6 and TNF-α), and reduced serum levels of liver transaminases. In addition, analysis of the liver specimens indicated that injection with the 1% pO_2_ secretome maximized the expression of the intermediate molecules of the PIP3/Akt and IL-6/STAT3 signaling pathways, all of which are known to promote liver regeneration.

**Conclusions:**

The data of this study suggest that the secretome of ASCs cultured under 1% pO_2_ has the highest liver reparative and regenerative potential of all the secretomes tested here.

**Electronic supplementary material:**

The online version of this article (doi:10.1186/s13287-017-0635-x) contains supplementary material, which is available to authorized users.

## Background

Liver transplantation is considered to be the only therapeutic option for advanced hepatic failure. Although it is very effective, liver transplantation includes a number of limitations, including the need for donors, higher cost, risks of perioperative complications, and lifelong requirement of immunosuppressant regimens and subsequent adverse effects [1]. Recently, researchers have turned their attention to stem cells to develop new therapies which could overcome the limitations of liver transplantation. However, stem cell-based therapy was also confronted by several limitations, mostly related to short lifespan, immune-mediated rejection, senescence-induced genetic instability, and possible malignant transformation of stem cells [2]. To address these limitations, several investigators introduced the concept of secretome therapy, wherein the secretome of the stem cells is exploited therapeutically. The secretome refers to the complex set of molecules secreted from stem cells or shed from their cell surfaces. The secretome has received much attention, since the discovery that principal mechanisms of stem cells are mediated by their secretome. The effects of the secretome have been demonstrated in many organ-specific diseases [3–8]. With relation to liver diseases, we have reported notable effects of the secretome in several rodent models of hepatic failure [9–12].

The composition of the secretome considerably depends on the culture conditions of stem cells. Therefore, obtaining a secretome of higher therapeutic potential is frequently accomplished by the addition of materials to the culture medium, such as growth factors, chemicals, or other clinically nonrelevant agents. However, the use of these materials can raise safety and cost concerns, especially when clinically applied. Hypoxic preconditioning (HP) is one of the culture-conditioning methods, wherein hypoxia, a physical stimulus, is used to stimulate stem cells to release the secretome, instead of chemical stimuli. Hence, HP is advantageous over chemical preconditioning methods in terms of safety and cost-effectiveness. In addition, HP is known to activate a number of signaling pathways prerequisite for survival, proliferation, and release of proinflammatory cytokines, such as the phosphatidylinositol-3,4,5-triphosphate (PIP3)/Akt, p38 mitogen-activated protein kinase (p38MAPK), and ERK pathways [13]. We have shown previously that a hypoxic-preconditioned secretome promotes the functional and regenerative capacity of the liver more effectively than a control secretome [10]. In this study, we aim to precisely determine the optimal oxygen partial pressure (pO_2_) in cell culture system that would activate the stem cells to release a secretome of highest liver reparative and regenerative capacity.

## Methods

### Cell culture

The AML12 mouse hepatocyte cell line was obtained from American Type Culture Collection (ATCC, Manassas, VA, USA). AML12 cells were maintained in Dulbecco’s Modified Eagle Medium/Ham’s F-12 (DMEM/F12; Thermo, Carlsbad, CA, USA). The medium was supplemented with 10% fetal bovine serum (FBS; GibcoBRL, Carlsbad, CA, USA), 1% antibiotics (Thermo), 1 × ITS supplement (Insulin–Transferrin–Selenium-G supplement; Invitrogen, Carlsbad, CA, USA), and 40 ng/ml dexamethasone (Sigma-Aldrich, St. Louis, MO, USA) at 37 °C. TCMK-1 cells (mouse kidney cells) were purchased from Korean Cell Line Bank (KCLB, Seoul, South Korea), and HK2 cells (human kidney cells) were kindly donated by Dr H.S. Hwang of the Catholic University of Korea. TCMK-1 and HK2 cells were maintained in DMEM (Thermo). The medium was supplemented with 10% FBS and 1% antibiotics at 37 °C. The adipose tissue-derived stem cells (ASCs) were kindly donated by Hurim BioCell Co. (Seoul, South Korea). ASCs were cultured in MesenPRO RS basal medium (GibcoBRL) supplemented with antibiotics (Antibiotic-Antimycotic; Invitrogen) at 37 °C. The cultured ASCs were shown to have characteristics of mesenchymal stem cells (MSCs); they expressed the MSC marker (CD90) and did not express hematopoietic markers (CD31 and CD34) [10].

### Establishment of ischemia–reperfusion injury in cell culture

To induce in-vitro ischemic injury, the AML12, TCMK-1, and HK2 cell lines were incubated in a Krebs–Henseleit buffer with 10 μM antimycin A and 1 mM 2-deoxyglucose for 1 h [14, 15]. Thereafter, reperfusion was achieved by washing the cells in Krebs–Henseleit buffer and then incubating in the complete growth medium (DMEM/F12) for 1 h.

### Preparation of secretome obtained under the different cultural pO_2_

ASCs were re-fed with serum-free low-glucose DMEM. ASCs were then cultured under either normoxic (21% pO_2_) or hypoxic (10%, 5%, and 1% pO_2_) conditions. The duration of HP was determined to be 24 h, during which the expression of signaling intermediates was highly amplified (Additional file 1: Figure S1). Hypoxic preconditioned conditioned media (CMs) were obtained by placing the ASCs in a hypoxic chamber (MIC-101; Billups-Rothenberg Inc., San Diego, CA, USA) at 37 °C for 24 h. Each CM was then concentrated 25-fold, using ultrafiltration units (Amicon Ultra-PL 3; Millipore, Bedford, MA, USA) with a 3-kDa cutoff, and the concentrated CM was considered the secretome. The secretomes with normoxic or various HP were stored at −80 °C until use.

### Cell proliferation assay

Cell proliferation was evaluated with 2-(4-iodophenyl)-3-(4-nitrophenyl)-5-(2,4-disulfophenyl)-2H-tetrazolium (water soluble tetrazolium salt (WST-1)) assay using the EZ-Cytox Cell Proliferation Assay kit (Itsbio, Seoul, Republic of Korea) according to the manufacturer’s instruction. Briefly, AML12 cells were cultured overnight (1 × 10^4^ cells per well) in 96-well plates. The 96-well plates were washed with phosphate-buffered saline (PBS) twice, and were incubated with the secretome of various cultural pO_2_. The reagent from the EZ-Cytox Cell Proliferation Assay kit was then applied to each well. Absorbance was measured at 450 nm using the microplate reader (model 680; Bio-Rad, CA, USA).

### Quantitative real-time PCR

Total RNA of ASCs was extracted using Tri-RNA reagent (Favorgen, Ping-Tung, Taiwan) according to the manufacturer’s instructions. Reverse transcription was performed with 1 μg of RNA, random primers, and M-MLV Reverse transcriptase (Promega, MI, USA). The primers used for SYBR Green reverse-transcription qPCR (RT-qPCR) were as follows: IL-6, forward 5′-CACACAGACAGCCACTCACC-3′ and reverse 5′-TTTTCTGCCAGTGCCTCTTT-3′; vascular endothelial growth factor (VEGF), forward 5′-TCTTCAAGCCATCCTGTGTG-3′ and reverse 5′-ATCTGCATGGTGATGTTGGA-3′; hepatocyte growth factor (HGF), forward 5′-TGCTGTCCTGGATGATTTTG-3′ and reverse 5′-AGTGTAGCCCCAGCCATAAA-3′; NAD-dependent deacetylase sirtuin-1 (SIRT1) [16], forward 5′-AGAACCACCAAAGCGGAAA-3′ and reverse 5′-TCCCACAGGAGACAGAAACC-3′; and GAPDH, forward 5′-GCACCGTCAAGGCTGAGAAC-3′ and reverse 5′-TGGTGAAGACGCCAGTGGA-3′. RT-qPCR was performed with the Applied Biosystems 7500 Fast Real-Time PCR System (Life technologies, Carlsbad, CA, USA). After normalizing to the *GAPDH* gene, expression levels for each target gene were calculated using the comparative threshold cycle (CT) method. Data are presented as the mean ± standard deviation (SD) from three independent experiments.

### Western blotting analysis

AML12 cells and liver specimens obtained from hepatectomized mice were lysed using the EzRIPA Lysis kit (ATTO Corporation; Tokyo, Japan), and quantified by Bradford reagent (Bio-Rad). Proteins were visualized by western blot analysis using the following primary antibodies (1:1000 dilution) at 4 °C overnight and then with HRP-conjugated secondary antibodies (1:2000 dilution) for 1 h at 25 °C: primary antibodies against proliferating cell nuclear antigen (PCNA), phosphor-signal transducer and activator of transcription 3 (p-STAT3), STAT3, HGF, VEGF, SIRT1, phosphor-serine/threonine-protein kinase (p-AKT), AKT, phosphor-extracellular signal-regulated kinases-(p-ERK), ERK, myeloid cell leukemia-1 (Mcl-1), bcl-2-like protein 4 (Bax), hypoxia-inducible factor-1α (HIF-1α), and β-actin. Horseradish peroxidase (HRP)-conjugated secondary antibodies were obtained from Cell Signaling Technology (Beverly, MA, USA). Specific immune complexes were detected using the Western Blotting Plus Chemiluminescence Reagent (Millipore).

### Injection of secretome with different cultural pO_2_ into the partially hepatectomized mice

Six-week-old male BALB/c mice (Samtako biokorea, Osan, South Korea) were used in this study. A partial hepatectomy (PH) was performed under tiletamine–zolazepam sedation (Zoletil 20®; Virbac, Nice, France) (30 mg/kg i.p.); the left lateral lobe (about 30% of the total liver mass) and the whole median lobe (about 40% of the total liver mass) were resected, leading to an approximately 70% reduction in liver mass. Subsequently, the mice were infused intravenously with the CM that had been obtained under the various cultural O_2_ tensions. Subsequently, the mice were intravenously infused with secretome with different cultural pO_2_. The mice were divided into five experimental groups, according to the materials administered: saline (0.1 ml normal saline), and secretome with 21%, 10%, 5%, and 1% cultural pO_2_. Each experimental group consisted of 25 mice (*N* = 125), and was further divided into two subgroups according to the manner of specimen collection. One subgroup (*n* = 5 in each group; *N* = 25) was for obtaining continuous data such as the levels of serum transaminases, and the other subgroup (*n* = 20 in each group; *N* = 100) was for obtaining data such as sera and liver specimens, after euthanizing the five mice per each group on day 1, 2, 3, and 7, respectively. The excised liver weights were utilized for the estimation of liver regeneration; LW/BW was calculated as the ratio (percentage) of liver weight to body weight [17–19].

### Immunohistochemical analysis

Paraffin-embedded tissue sections were deparaffinized in xylene and rehydrated in a graded series of alcohol. The antigen was retrieved with 0.01 M citrate buffer (pH 6.0) by heating the sample in microwave for 10 min. The tissue sections were then placed in 3% hydrogen peroxide for 5 min to inactivate the endogenous peroxidase, and blocked for 30 min with normal horse serum (DakoCytomation LSAB2 System-HRP kit; DakoCytomation, Glostrup, Denmark). The primary antibodies used for this study were Ki-67 rabbit polyclonal antibody (1:300; Abcam, Cambridge, MA, USA), VEGF and SIRT1 mouse polyclonal antibodies (1:150; all from Abcam), cleaved caspase-3 rabbit polyclonal antibody (1:300; Cell Signaling), and B-cell lymphoma-extra large (Bcl-xL) mouse monoclonal antibodies (1:100: Santa Cruz biotechnology, Dallas, TX, USA). The prediluted primary antibodies were applied overnight at 4 °C. The slides were then treated with biotinylated secondary antibody for 30 min at room temperature, followed by the treatment with streptavidin-HRP and 3,3′-diaminobenzidine solution for another 10 min at room temperature. Tissue sections were counterstained with hematoxylin.

### Enzyme-linked immunosorbent assay

The serum levels of IL-6 and TNF-α of the partially hepatectomized mice were determined by an enzyme-linked immunosorbent assay (ELISA) kit (eBioscience, San Diego, CA, USA) on day 7 after injection according to the manufacturer’s instructions.

### Assessment of liver functions

Blood samples were obtained from each mouse, centrifuged for 10 min at 10,000 rpm, and serum collected on day 1, 2, 3, and 7 after injection. The parameters for liver injury, such as aspartate transaminase (AST) and alanine transaminase (ALT), were measured using an IDEXX VetTest Chemistry Analyzer (IDEXX Laboratories, Westbrook, ME, USA).

### Statistical analysis

All data were analyzed using SPSS 11.0 software (SPSS, Chicago, IL, USA) and are presented as the mean ± SD. The Mann–Whitney *U* test was used for the mean comparison of two groups, and the Kruskal–Wallis test was used for the comparison of three or more groups. *p* < 0.05 was considered statistically significant.

## Results

### Effects of various concentrations of culture pO_2_ on the microenvironment of ASCs

**Fig. 1:**
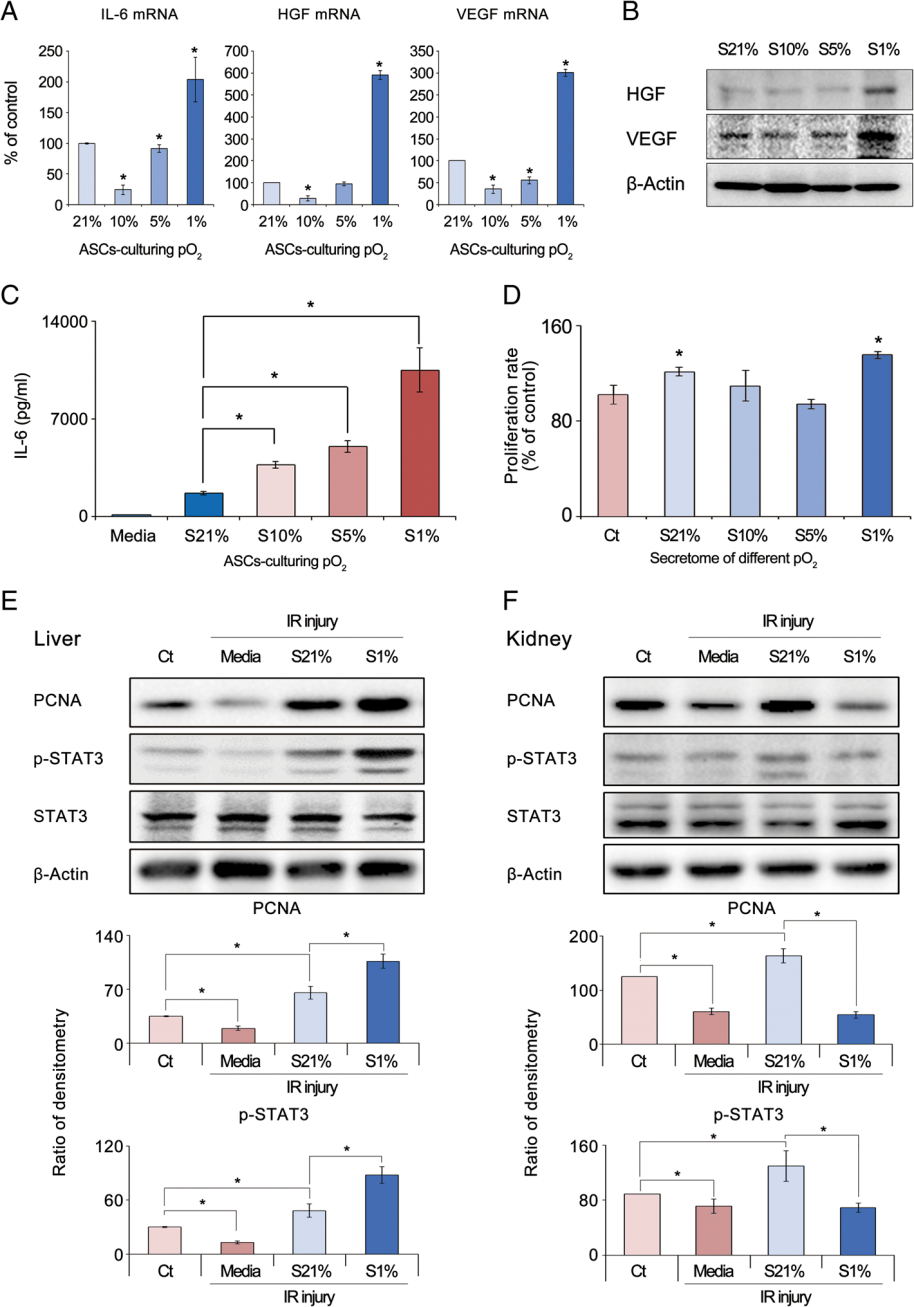
Effects of various concentrations of culture pO_2_ on the microenvironment of ASCs. **a** Real-time RT-PCR showed mRNA expression of the markers for liver regeneration (IL-6, HGF, and VEGF) according to different concentrations of culture pO_2_. mRNA expression of these markers was significantly higher in AML12 cells cultured under 1% pO_2_ than in AML12 cells cultured under other pO_2_ concentrations. **b** Western blot analysis showed highest expression of HGF and VEGF in the 1% pO_2_ group. **c** ELISA showed the highest concentration of IL-6 in the 1% pO_2_ group. **d** Effects of secretome cultured under 21%, 10%, 5%, and 1% pO_2_ on proliferation of AML12 hepatocytes. AML12 cells cultured with secretome of 1% pO_2_ showed the highest cell proliferation, followed by cells cultured with secretome of 21%, 10%, and 5% pO_2_, in that order. **e** Effects of secretome with culture 1% pO_2_ on injured AML12 hepatocytes. Western blot analysis showed that supplementation with 1% pO_2_ secretome significantly increased expression levels of PCNA and p-STAT3 better than 21% pO_2_ secretome. **f** Effects of the secretome with culture 1% pO_2_ on injured TCMK-1 renal cells. Western blot analysis showed that supplementation with 21% pO_2_ secretome significantly increased the expression levels of PCNA and p-STAT3 better than the 1% pO_2_ secretome. Data show mean and SD for three independent experiments. *p < 0.05 compared to control. *ASC* adipose-derived stem cell, *Ct* control, *HGF* hepatocyte growth factor, *IL* interleukin, *IR* ischemia–reperfusion, *PCNA* proliferating cell nuclear antigen, *pO*_*2*_ oxygen partial pressure, *p-STAT3* phospho-signal transducer and activator of transcription 3, *S21*% secretome of culture 21% pO_2_, *S10*% secretome of culture 10% pO_2_, *S5*% secretome of culture 5% pO_2_, *S1*% secretome of culture 1% pO_2_, *VEGF* vascular endothelial cell growth factor

Although HP is advantageous for promoting cell proliferation, an optimized concentration of pO_2_ for attaining a secretome with higher therapeutic potential has not been determined. We thus compared mRNA expression of the markers for liver regeneration (IL-6, HGF, and VEGF) according to the different concentrations of culture pO_2_ by real-time RT-PCR (Fig. 1a). The mRNA expression of these markers was significantly higher in AML12 cells cultured under 1% pO_2_ than in the AML12 cells cultured under other pO_2_ concentrations (*p <* 0.05). In addition, the 1% pO2 group showed the highest expression of HGF and VEGF in western blot analysis (Fig. 1b), and the highest concentration of IL-6 tested by ELISA (Fig. 1c).

Subsequently, we compared the effects of secretome cultured under 21%, 10%, 5%, and 1% pO_2_ on the proliferation of AML12 hepatocytes (Fig. 1d). The AML12 cells cultured with the secretome of 1% pO_2_ showed the highest cell proliferation, followed by the cells cultured with the secretome of 21%, 10%, and 5% pO_2_, in that order.

### Effects of the secretome with culture 1% pO_2_ on injured hepatocytes or renal cells

We then investigated the effects of the secretome with culture 1% pO_2_ on the injured hepatocytes or renal cells. After establishing in-vitro ischemia–reperfusion (IR)-injured AML12 hepatocytes or HK2 renal cells, we investigated the effects of the secretome with culture 1% pO_2_ on the expression of the proliferation markers (STAT3 and PCNA) in these cells. In the IR-injured AML12 hepatocytes, supplementation with the 1% pO_2_ secretome significantly increased the expression levels of PCNA and p-STAT3 better than the 21% pO_2_ secretome (*p <* 0.05) (Fig. 1e). However, in the IR-injured HK2 renal cells, supplementation with the 1% pO_2_ secretome did not significantly increase, but rather decreased, the expression levels of PCNA and p-STAT3 compared to supplementation with the 21% pO_2_ secretome (Fig. 1f). In the IR-induced human renal cells (HK2 cells), there was no significant difference in the expression of these markers between the two different pO_2_ secretome groups (Additional file 2: Figure S2). The data presented here suggest that the therapeutic effect of the secretome could be tissue specific; the secretome with culture 1% pO_2_ was more advantageous in repairing injured hepatocytes than the injured renal cells.

### Effects of the secretome with different concentrations of culture pO_2_ on hepatic recovery in partially hepatectomized mice

**Fig. 2:**
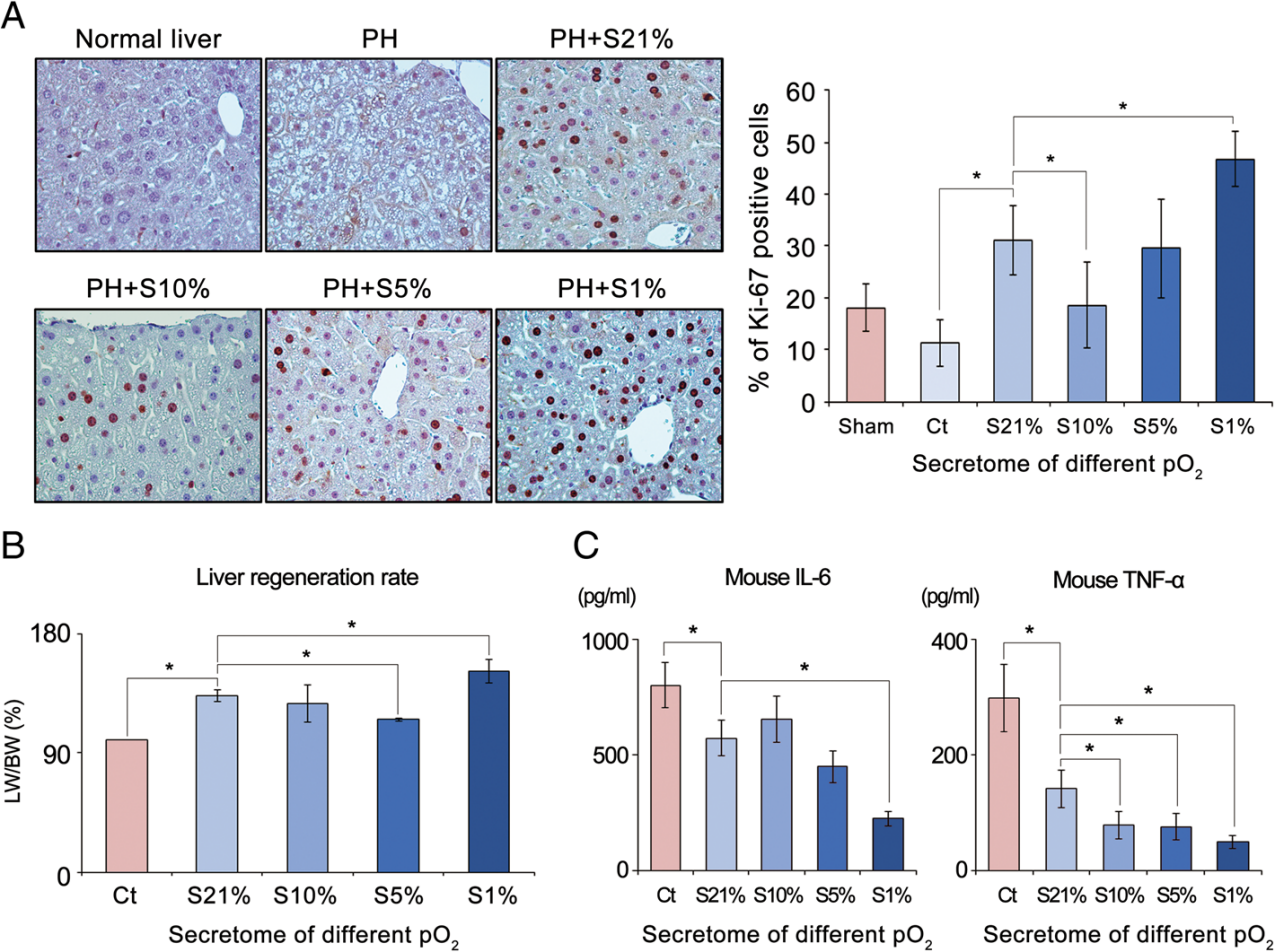
Effects of the secretome with different concentrations of culture pO_2_ on hepatic recovery in partially hepatectomized mice. **a** (*Left*) Ki67 immunohistochemistry of liver specimens obtained from the mice injected with the secretome with different concentrations of culture pO_2_ at day 2 after PH. (*Right*) Percentage of Ki67-positive cells in each group at day 2 after injection. Of the secretome-injected groups, the number of Ki67-positive cells was highest in the liver specimens injected with the secretome of culture 1% pO_2_, followed by that with culture 21%, 5%, and 10% pO_2_, in that order. **b** Estimation of liver regeneration rates by LW/BW in each group at day 7 after injection. Of the secretome-injected groups, injection with the secretome of 1% pO_2_ exhibited the highest LW/BW, followed by injection with the secretome of 21%, 10%, and 5% pO_2_, in that order. **c** ELISA for the serum levels of IL-6 and TNF-α in each group at day 2 after injection. Of the secretome-injected groups, the serum levels of IL-6 and TNF-α were most significantly decreased in the 1% pO_2_ secretome group. Each group included five mice (25 mice in total). Data show means and SD for three independent experiments. *p < 0.05 compared to control. *Ct* control, *IL* interleukin, *LW/BW* ratio of liver weight to body weight, *PH* partial hepatectomy, *pO*_*2*_ oxygen partial pressure, *S21*% secretome of culture 21% pO_2_, *S10*% secretome of culture 10% pO_2_, *S5*% secretome of culture 5% pO_2_, *S1*% secretome of culture 1% pO_2_, *TNF*-α tumor necrosis factor alpha

After performing 70% PH, the mice were injected with the secretome containing different concentrations of culture pO_2_ (1%, 5%, 10%, and 21%), respectively. We then determined the effects of the secretome with different concentrations of culture pO_2_ on liver regeneration using both Ki67 immunohistochemistry and liver weight measurement (LW/BW). Antigen Ki67 is a nuclear protein associated with the transcription of ribosomal RNA and therefore is exclusively expressed in proliferating cells [20]. Secretome-injected groups showed a higher number of Ki67-positive cells than the control group on day 2 after injection (*p <* 0.05) (Fig. 2a). Of the secretome-injected groups, the number of Ki67-positive cells was the highest in the liver specimens injected with the secretome of culture 1% pO_2_, followed by that with culture 21%, 5%, and 10% pO_2_, in that order (*p <* 0.05). The liver regeneration rate was also assessed in terms of LW/BW on day 7 after injection (Fig. 2b). Of the secretome-injected groups, injection with the secretome of 1% pO_2_ exhibited the highest LW/BW (151% compared to the control), followed by injection with the secretome of 21%, 10%, and 5% pO_2_, in that order (*p <* 0.05).

The secretome has the potential of reducing systemic inflammation [11, 12, 21–23]. We thus compared the effects of the secretome with different concentrations of culture pO_2_ on the serum concentration of representative proinflammatory cytokines (IL-6 and TNF-α) on day 2 after injection (Fig. 2c). The serum levels of IL-6 and TNF-α were significantly decreased in all secretome-injected groups compared with the control group (*p <* 0.05). Of the secretome-injected groups, the serum levels of IL-6 were most significantly decreased in the 1% pO_2_ secretome group, followed by the secretome groups with 5%, 21%, and 10% pO_2_, in that order (*p <* 0.05). Similarly, the serum levels of TNF-α were most significantly decreased in the 1% pO_2_ secretome group (*p <* 0.05).

### Effects of the secretome with different concentrations of culture pO_2_ on hepatic function in partially hepatectomized mice

**Fig. 3:**
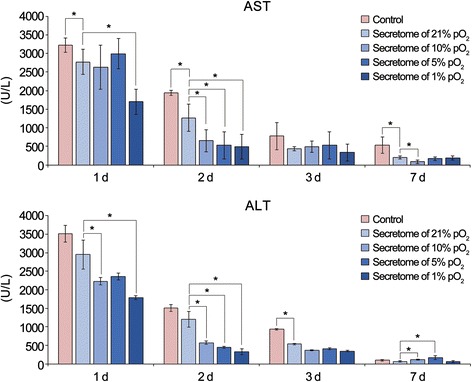
Effects of the secretome with different concentrations of culture pO2 on hepatic function in partially hepatectomized mice. Mouse serum samples were collected on day (*d*) 1, 2, 3, and 7 after injection, and serum levels of AST and ALT were determined. Of all the secretome-injected groups, the 1% pO_2_ secretome group appeared to have the lowest levels of AST and ALT on most days (*p <* 0.05). Each group included five mice (25 mice in total). Data represent mean ± SD. *p < 0.05 compared to control. *ALT* alanine transaminase, *AST* aspartate transaminase, *pO*_*2*_ oxygen partial pressure

We investigated the effects of the secretome with different concentrations of culture pO_2_ on the serum levels of liver enzymes (AST and ALT) (Fig. 3). Mouse serum samples were collected on day 1, 2, 3, and 7 after injection, and the serum levels of AST and ALT were determined. Secretome-injected groups exhibited lower levels of AST and ALT than the control group (*p <* 0.05). Of all the secretome-injected groups, the 1% pO_2_ secretome group appeared to have the lowest levels of AST and ALT on most days (*p <* 0.05).

### Effects of the secretome with different concentrations of culture pO_2_ on the signaling pathways essential for liver regeneration in the mouse liver

**Fig. 4:**
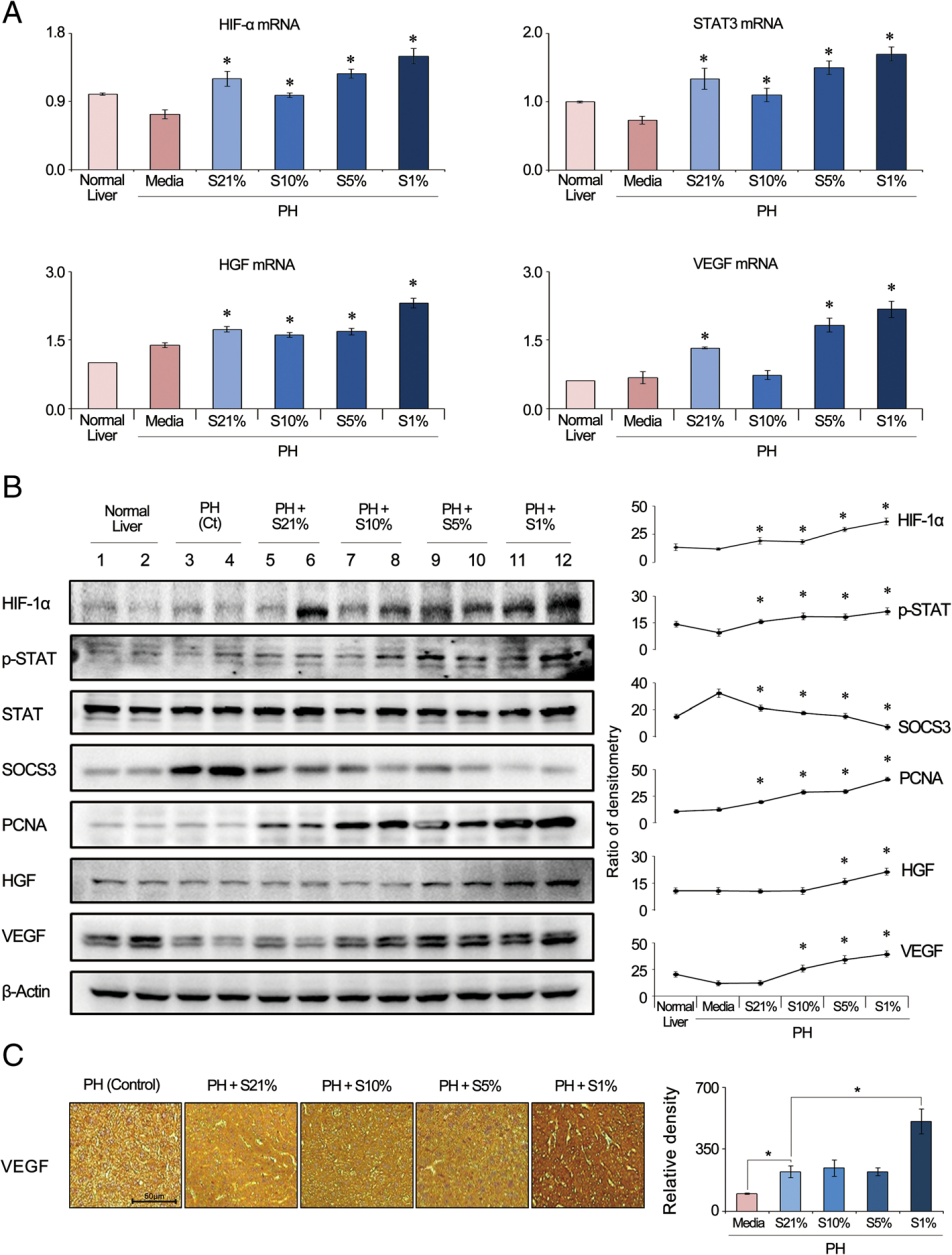
Effects of the secretome with different concentrations of culture pO_2_ on the signaling pathways essential for cell proliferation in the mouse liver. **a** Real-time RT-PCR showing mRNA expression of HIF-1α, STAT3, HGF, and VEGF in the liver specimens of the secretome-injected mice on day 2 after injection. Of the groups, the 1% pO_2_ group showed the highest mRNA expression of HGF (*left*) and VEGF (*right*). **b** (*Left*) Western blot analysis of the markers for liver cell proliferation in the mouse liver specimens on day 2 after injection. (*Right*) Relative densities of each marker. The 1% pO_2_ secretome group showed the highest expression of HIF-1α, PCNA, HGF, and VEGF. The 1% pO_2_ secretome group showed the highest expression of p-STAT3 as well as the lowest expression of SOCS3 (a key negative regulator of IL-6/STAT3 signaling). **c** VEGF immunohistochemistry of liver specimens demonstrating the highest expression of VEGF in the 1% pO_2_ secretome group on day 2 after injection. Data represent mean ± SD. *p < 0.05 compared to control. *Ct* control, *HGF* hepatocyte growth factor, *HIF-1α* hypoxia-inducible factor-1α, *PCNA* proliferating cell nuclear antigen, *PH* partial hepatectomy, *S21*% secretome of culture 21% pO_2_, *S10*% secretome of culture 10% pO_2_, *S5*% secretome of culture 5% pO_2_, *S1*% secretome of culture 1% pO_2_, *SOCS3* suppressor of cytokine signaling 3, *p-STAT3* phospho-signal transducer and activator of transcription 3, *VEGF* vascular endothelial growth factor

We investigated the effects of the secretome with different concentrations of culture pO_2_ on the markers for cell proliferation in the mouse liver on day 2 after injection. In real-time RT-PCR, the 1% pO_2_ secretome group exhibited the highest mRNA expression levels of HIF-1α, STAT3, HGF, and VEGF (Fig. 4a). Western blot analysis also revealed that the 1% pO_2_ secretome group showed the highest expression of the markers for liver cell proliferation, including PCNA, HGF, and VEGF, as well as HIF-1α (Fig. 4b). The 1% pO_2_ secretome group showed the highest expression of p-STAT3 as well as the lowest expression of SOCS3, a key negative regulator of IL-6/STAT3 signaling. VEGF immunohistochemistry of the liver specimens also confirmed that the 1% pO_2_ secretome group exhibits the highest expression of VEGF (Fig. 4c).

**Fig. 5:**
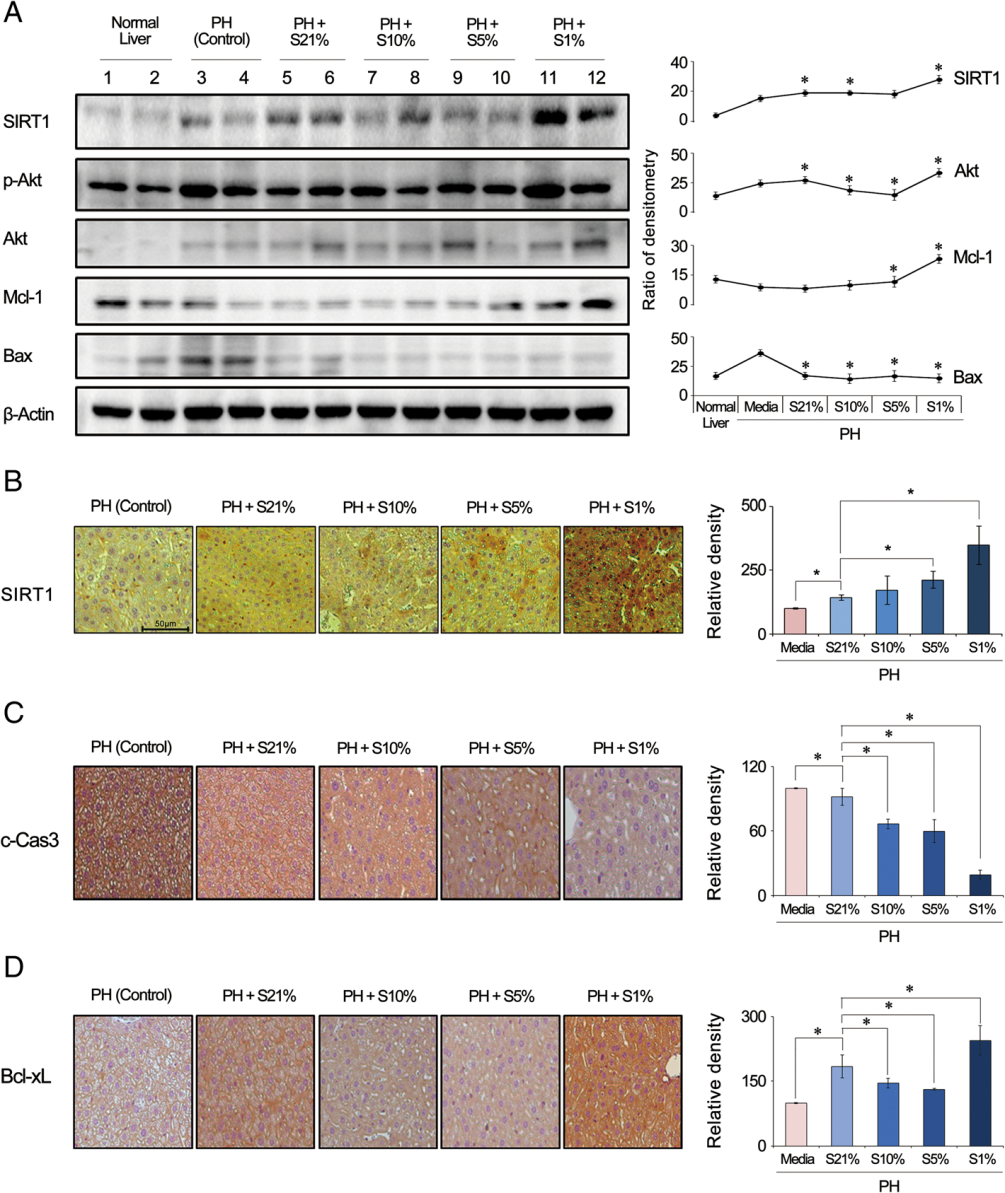
Effects of the secretome with different concentrations of culture pO_2_ on signaling pathways essential for hypertrophy in the mouse liver. **a** (*Left*) Western blot analysis of the markers for liver cell hypertrophy in the mouse liver specimens on day 2 after injection. (*Right*) Relative densities of each marker. Of the groups, the 1% pO_2_ secretome group showed the highest expression of SIRT1 and Akt. The 1% pO_2_ secretome group also exhibited the lowest expression of Bax (a pro-apoptotic marker) as well as the highest expression of Mcl-1 (an anti-apoptotic marker). **b** SIRT1 immunohistochemistry of liver specimens demonstrating the highest expression of SIRT1 in the 1% pO_2_ secretome group on day 2 after injection. **c** Cleaved caspase-3 immunohistochemistry of liver specimens demonstrating the lowest expression of cleaved caspase-3 in the 1% pO_2_ secretome group on day 2 after injection. **d** Bcl-xL immunohistochemistry of liver specimens demonstrating the highest expression of Bcl-xL in the 1% pO_2_ secretome group on day 2 after injection. Data represent mean ± SD. *p < 0.05 compared to control. *Bax* bcl-2-like protein 4, *Bcl-xL* B-cell lymphoma—extra large, *c-Cas3* cleaved caspase-3, *Mcl-1* myeloid cell leukemia-1, *PH* partial hepatectomy, *SIRT1* sirtuin 1, *S21*% secretome of culture 21% pO_2_, *S10*% secretome of culture 10% pO_2_, *S5*% secretome of culture 5% pO_2_, *S1*% secretome of culture 1% pO_2_

Subsequently, we investigated the effects of the secretome with different concentrations of culture pO_2_ on the markers for hypertrophy in the mouse liver on day 2 after injection. Western blot analysis revealed that the 1% pO_2_ secretome group showed the highest expression of SIRT1 as well as Akt (Fig. 5a). In addition, the 1% pO_2_ secretome group exhibited the lowest expression of Bax (a pro-apoptotic marker) as well as the highest expression of Mcl-1 (an anti-apoptotic marker). SIRT1 immunohistochemistry of the liver specimens confirmed that the 1% pO_2_ secretome group exhibited the highest expression of SIRT1 (Fig. 5b). In addition, immunohistochemistry of the liver specimens also validated that the 1% secretome group exhibited the lowest expression of the pro-apoptotic marker (c-caspase 9) and the highest expression of the anti-apoptotic marker (Bcl-xL) (Fig. 5c, d). Taken altogether, the data presented here suggest that the 1% pO_2_ secretome group has the highest liver regenerative and anti-apoptotic potential among the secretomes tested with different concentrations of culture pO_2_.

## Discussion

In this study, we intended to precisely determine the optimal pO_2_ in a cell culture system that causes ASCs to release the secretome with the highest reparative and regenerative capacity in the liver. Hepatocytes cultured under 1% pO_2_ showed the highest expression of proliferation-associated markers such as IL-6, HGF, and VEGF. The AML12 cells cultured with the secretome of 1% pO_2_ showed the highest cell proliferation, followed by the cells cultured with the secretome of 21%, 10%, and 5% pO_2_, in that order. When the secretomes of various culture pO_2_ were injected into partially hepatectomized mice, the 1% pO_2_ secretome most significantly increased liver regeneration and reduced serum levels of proinflammatory mediators (IL-6 and TNF-α) and liver transaminases. In addition, analysis of the mouse liver specimens indicated that injection with 1% pO_2_ secretome maximized expression of the essential intermediates in the PIP3/Akt and IL-6/STAT3 signaling pathways, all of which are known to promote liver regeneration. Taken together, our experiment showed that, of the various culture pO_2_ (1%, 5%, 10%, and 21%), 1% pO_2_ was most advantageous for obtaining the secretome that maximizes liver regenerative and reparative potential.

Several lines of evidence indicated that stem cells proliferate better under hypoxic condition than under normoxia [24–27]. For instance, Grayson et al. [25] showed that human MSCs under 2% pO_2_ exhibited 30-fold increase of proliferation for seven passages compared to MSCs under normoxia. Lennon et al. [26] also showed that culture of rat MSCs under 5% pO_2_ resulted in approximately 40% higher cell number at first passage than culture under normoxia. In addition, reduced pO_2_ was found to decrease the population doubling time of marrow-isolated adult multilineage inducible cells, with 3% pO_2_ showing the maximum effect [24]. Although the underlying mechanism is still unknown, it could be greatly attributed to the fact that the actual in-vivo pO_2_ is considerably low, ranging from 1% (deep zone of cartilage) to 12% (blood) [28]. Similar to other internal organs, the liver maintains low oxygen tension, ranging from 3% (perivenous area) to 7% (periportal area) [29]. Our study suggests that, of the various culture pO_2_ (1%, 5%, 10%, and 21%), 1% pO_2_ is most advantageous to stimulate ASC proliferation.

Until now, several explanations have been provided to clarify the reason why HP is beneficial to cell growth. Some authors insisted that HP extends the survival of cultured cells through a HIF-1α-mediated mechanism. They found that hypoxia activates a complex array of signaling pathways favoring the stabilization of HIF-1α, which otherwise would be degraded [13]. Stabilized HIF-1α binds and activates promoter regions of hypoxia-responsive genes, whose expression contributes to survivability of MSCs by promoting the expression of glucose-6-phosphate transporter. The target genes of HIF-1α also include those encoding pro-angiogenetic factors, such as IL-6 and VEGF. Moreover, hypoxia-induced activation of Akt/p38MAPK also leads to upregulation of anti-apoptotic proteins such as Bcl-2 and Bcl-xL [30]. The upregulated anti-apoptotic proteins in turn reduce apoptosis by decreasing the expression of pro-apoptotic proteins such as Bax and Caspase-3.

The fundamental concept of our study is that HP has the potential of upregulating secretome release from MSCs [30–32]. The secretome elicited by HP is known to have immunomodulatory properties such as inhibition of the activities of peripheral blood mononuclear cells and promotion of inflammation via cytokines [30]. Ohnishi et al. [33] reported that culturing rat MSCs with 1% pO_2_ for 24 h increased the expression of a number of genes related to cell proliferation and survival, including VEGF-D, heparin-binding epidermal growth factor, matrix metalloproteinase-9, and placental growth factor. In another study, culturing human MSCs with 1% pO_2_ for 48 h increased the secretion of VEGF, decreased the levels of transforming growth factor-β1 and basic fibroblast growth factor (but insignificantly), and did not change the levels of IL-6, IL-8, and tissue inhibitor of metalloproteinases-1 [31]. Thus, it appears that HP can selectively increase, decrease, or have no impact on certain components of the secretome. In addition, HP can increase migration and homing abilities of MSCs by upregulating the expression of CX3XR1 and CXCR4, as well as cMet (receptor for hepatocyte growth factor) [34–36].

In our study, SIRT1 expression was highest in the mouse liver in which the secretome of the lowest (1%) culture pO_2_ was infused. SIRT1 deacetylates a number of essential transcriptional regulatory proteins, and thereby regulates a variety of physiological processes, including stress responses, metabolism, apoptosis, calorie restriction, and aging [37, 38]. In the liver, SIRT1 functions as an essential regulator of metabolic processes, such as gluconeogenesis, fatty acid beta-oxidation, mitochondrial activity, and cholesterol flux, all of which occur in response to an intracellular rise in the NAD^+^/NADH ratio when energy supplies are low [39]. Accumulating evidence indicates that SIRT1 is considerably involved in the liver regeneration [40–43]. Bellet et al. [43] showed that the higher expression of SIRT1 directly facilitates liver regeneration by promoting G1/S transition and fatty acid beta-oxidation which is essential for liver regeneration.

**Fig. 6:**
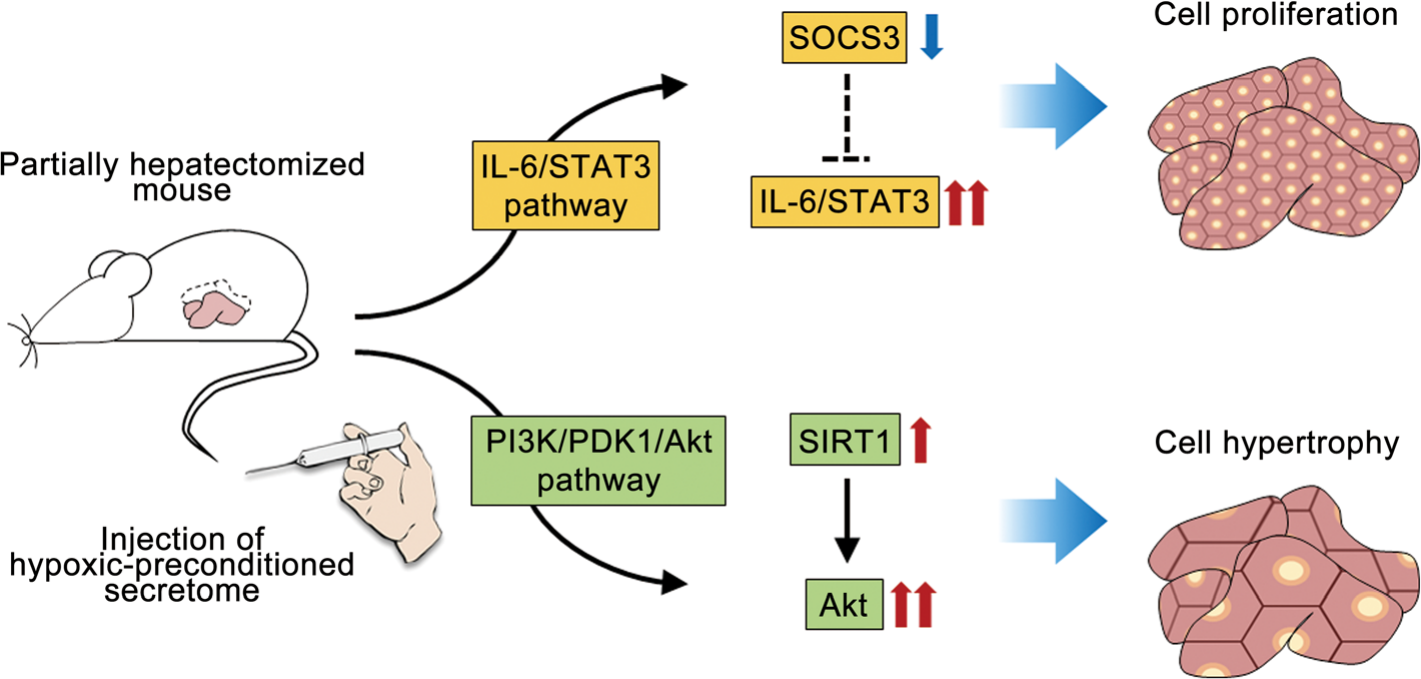
Proposed mechanism of hypoxic-conditioned secretome effects on liver regeneration. Liver regeneration is essentially achieved by the combination of two distinct factors: cell proliferation (increase in the number of hepatocytes) and hypertrophy (increase in the size of hepatocytes). Cell proliferation is mediated by the IL-6/STAT3 pathway, and hypertrophy is mediated by the PI3K/PDK/Akt pathway. We provide two independent mechanisms by which hypoxic-conditioned secretome improves liver regeneration. First, hypoxic-preconditioned secretome promotes liver cell proliferation by persistent and uninhibited expression of STAT3 in the liver which is caused by decreased expression of SOCS3. Second, hypoxic-preconditioned secretome promotes liver cell hypertrophy by upregulating Akt expression which is activated by higher expression of SIRT1. *IL* interleukin, *PDK1* 3-phosphoinositide-dependent protein kinase 1, *PI3K* phosphoinositide 3-kinase, *STAT3* signal transducers and activators of transcription 3, *SIRT1* sirtuin 1, *SOCS3* suppressor of cytokine signaling 3

Liver regeneration is essentially achieved by the combination of two distinct factors: cell proliferation (increase in the number of hepatocytes) and hypertrophy (increase in the size of hepatocytes). It has been identified that cell proliferation is mediated by the IL-6/STAT3 pathway, and hypertrophy is mediated by the PI3-K/PDK/Akt pathway [44]. We previously showed that the secretome obtained from HP promotes liver regeneration by persistent and uninhibited expression of STAT3 in the liver which is caused by decreased expression of SOCS3 [10]. In this study, apart from the HP effects on the IL-6/STAT3 pathway, we investigated the HP effects on the PI3-K/PDK/Akt pathway. The group of 1% pO_2_ secretome showed the highest expression of SIRT1 in the liver specimens. This could be explained by a previous study which demonstrated that acute hypoxia increases the SIRT1 expression in a HIF-dependent manner [45]. The group of 1% pO_2_ secretome also showed the highest expression of Akt in the liver specimens. Recent advancements in cell biology have also identified sirtuins as major regulators of Akt activation [46]. Of the sirtuins, SIRT1 was discovered to directly deacetylate Akt, thereby enabling its binding to phosphatidylinositol (3,4,5)-triphosphate (PI3P). The binding between Akt and PI3P results in a conformational change that exposes the kinase domain of Akt for phosphorylation and activation by 3-phosphoinositide-dependent protein kinase 1 (PDK1). Taken altogether, we think that the 1% pO_2_ secretome induces the highest liver regenerative potential through the activation of both the IL-6/STAT3 and the PI3K/PDK/Akt pathways (Fig. 6).

In our study, hepatocytes responded more effectively with hypoxic secretome than the kidney cells, thereby resulting in higher expression of the proliferation maker (PCNA) and the intermediates. These differences may be due to the specific characteristics of hepatocytes. Unlike other cells, hepatocytes are capable of responding positively to damage through matrix turnover or remodeling, rather than simply being the targets of insults or bystanders in the process of injury. The ability to cope with such insults is evidenced by improved regenerative potential of hepatocytes. In transplantation experiments using fumarylacetoacetate hydrolase (FAH)-deficient mice, hepatocytes have proved to have the capability of regenerating more than 70 times [47]. Thus, although hepatocytes appear to be quiescent in normal liver, they exhibit enormous proliferation potential when they are stimulated. We believe that such characterization of hepatocytes is the reason why hypoxic secretome treatment was more effective in hepatocytes than in kidney cells.

## Conclusions

Of all the secretomes cultured at different pO_2_ in this study, the 1% pO_2_ secretome appears to be optimum in cell culture, causing stem cells to release the secretome with the highest reparative and regenerative capacity in the liver. When the secretomes of various culture pO_2_ were injected into partially hepatectomized mice, the 1% pO_2_ secretome most significantly increased liver regeneration, reduced serum levels of proinflammatory cytokines, and reduced the elevated levels of liver enzymes. We can conclude that, of the various culture pO_2_ (1%, 5%, 10%, and 21%), 1% pO_2_ was the most advantageous for obtaining the secretome that maximizes liver regenerative and reparative potential. Therefore, we strongly recommend 1% HP of stem cells as an efficient and safe way of maximizing the liver-regenerative potential of secretome.

## Additional files

Additional file 1: Figure S1. Western blot analysis showing the comparison of the expression of various signaling mediates between 24-h and 48-h culturing periods. Expression of HIF-1α, p-Akt, p-STAT3, and p-ERK was further increased after 24-h incubation, leading us to decide 24-h hypoxic culturing of stem cells. (TIF 1429 kb)

Additional file 2: Figure S2. Western blot analysis showing the effects of the secretome with 21% and 1% pO_2_ on the IR-induced human renal cells (HK2 cells), respectively. There was no significant difference in the expression of these markers between the two different pO_2_ secretome groups. (TIF 1830 kb)

## Abbreviations

AKT: Serine/Threonine-protein kinase
ALT: Alanine transaminase
AST: Aspartate transaminase
ASC: Adipose tissue-derived stem cell
ATCC: American Type Culture Collection
CM: Conditioned medium
Bax: bcl-2-like protein 4
Bcl-xL: B-cell lymphoma—extra large
c-Cas3: Cleaved caspase-3
ERK: Extracellular signal-regulated kinase
FBS: Fetal bovine serum
HGF: Hepatocyte growth factor
HIF-1α: Hypoxia-inducible factor-1α
HP: Hypoxic preconditioning
Mcl-1: Myeloid cell leukemia-1
MSC: Mesenchymal stem cell
PIP3: Phosphatidylinositol-3,4,5-triphosphate
p38MAPK: p38 mitogen-activated protein kinase
PBS: Phosphate-buffered saline
PCNA: Proliferating cell nuclear antigen
p-STAT3: Phospho-signal transducer and activator of transcription 3
RT-qPCR: Reverse-transcription qPCR
SD: Standard deviation
SIRT1: NAD-dependent deacetylase sirtuin-1
VEGF: Vascular endothelial growth factor

## References

[1] Wertheim JA, Petrowsky H, Saab S, Kupiec-Weglinski JW, Busuttil RW (2011). Major challenges limiting liver transplantation in the United States. Am J Transplant.

[2] Baglio SR, Pegtel DM, Baldini N. Mesenchymal stem cell secreted vesicles provide novel opportunities in (stem) cell-free therapy. Front Physiol. 2012;3:359. 10.3389/fphys.2012.00359 PMC3434369 22973239

[3] Ahmadi M, Rahbarghazi R, Aslani MR, Shahbazfar AA, Kazemi M, Keyhanmanesh R (2017). Bone marrow mesenchymal stem cells and their conditioned media could potentially ameliorate ovalbumin-induced asthmatic changes. Biomed Pharmacother.

[4] Deng K, Lin DL, Hanzlicek B, Balog B, Penn MS, Kiedrowski MJ (2015). Mesenchymal stem cells and their secretome partially restore nerve and urethral function in a dual muscle and nerve injury stress urinary incontinence model. Am J Physiol Renal Physiol.

[5] Gazdhar A, Grad I, Tamo L, Gugger M, Feki A, Geiser T (2014). The secretome of induced pluripotent stem cells reduces lung fibrosis in part by hepatocyte growth factor. Stem Cell Res Ther.

[6] Khanabdali R, Rosdah AA, Dusting GJ, Lim SY (2016). Harnessing the secretome of cardiac stem cells as therapy for ischemic heart disease. Biochem Pharmacol.

[7] Shree N, Bhonde RR. Conditioned media from adipose tissue derived mesenchymal stem cells reverse insulin resistance in cellular models. J Cell Biochem. 2017;118:2037-2043. 10.1002/jcb.25777 27791278

[8] Teixeira FG, Carvalho MM, Panchalingam KM, Rodrigues AJ, Mendes-Pinheiro B, Anjo S (2017). Impact of the secretome of human mesenchymal stem cells on brain structure and animal behavior in a rat model of Parkinson’s disease. Stem Cells Transl Med.

[9] Lee SC, Jeong HJ, Lee SK, Kim SJ (2015). Lipopolysaccharide preconditioning of adipose-derived stem cells improves liver-regenerating activity of the secretome. Stem Cell Res Ther.

[10] Lee SC, Jeong HJ, Lee SK, Kim SJ (2016). Hypoxic conditioned medium from human adipose-derived stem cells promotes mouse liver regeneration through JAK/STAT3 signaling. Stem Cells Transl Med.

[11] Lee SC, Kim JO, Kim SJ (2015). Secretome from human adipose-derived stem cells protects mouse liver from hepatic ischemia-reperfusion injury. Surgery.

[12] Lee SK, Lee SC, Kim SJ (2015). A novel cell-free strategy for promoting mouse liver regeneration: utilization of a conditioned medium from adipose-derived stem cells. Hepatol Int.

[13] Kanichai M, Ferguson D, Prendergast PJ, Campbell VA (2008). Hypoxia promotes chondrogenesis in rat mesenchymal stem cells: a role for AKT and hypoxia-inducible factor (HIF)-1alpha. J Cell Physiol.

[14] Wang LT, Chen BL, Wu CT, Huang KH, Chiang CK, Hwa LS (2013). Protective role of AMP-activated protein kinase-evoked autophagy on an in vitro model of ischemia/reperfusion-induced renal tubular cell injury. PLoS One.

[15] Xie J, Guo Q (2006). Apoptosis antagonizing transcription factor protects renal tubule cells against oxidative damage and apoptosis induced by ischemia-reperfusion. J Am Soc Nephrol.

[16] Kanfi Y, Peshti V, Gozlan YM, Rathaus M, Gil R, Cohen HY (2008). Regulation of SIRT1 protein levels by nutrient availability. FEBS Lett.

[17] Bockhorn M, Goralski M, Prokofiev D, Dammann P, Grunewald P, Trippler M (2007). VEGF is important for early liver regeneration after partial hepatectomy. J Surg Res.

[18] Lehmann K, Tschuor C, Rickenbacher A, Jang JH, Oberkofler CE, Tschopp O (2012). Liver failure after extended hepatectomy in mice is mediated by a p21-dependent barrier to liver regeneration. Gastroenterology.

[19] Yu J, Yin S, Zhang W, Gao F, Liu Y, Chen Z (2013). Hypoxia preconditioned bone marrow mesenchymal stem cells promote liver regeneration in a rat massive hepatectomy model. Stem Cell Res Ther.

[20] Bullwinkel J, Baron-Luhr B, Ludemann A, Wohlenberg C, Gerdes J, Scholzen T (2006). Ki-67 protein is associated with ribosomal RNA transcription in quiescent and proliferating cells. J Cell Physiol.

[21] Aggarwal S, Pittenger MF (2005). Human mesenchymal stem cells modulate allogeneic immune cell responses. Blood.

[22] Hoogduijn MJ, Crop MJ, Peeters AM, Van Osch GJ, Balk AH, Ijzermans JN (2007). Human heart, spleen, and perirenal fat-derived mesenchymal stem cells have immunomodulatory capacities. Stem Cells Dev.

[23] Krampera M, Pasini A, Pizzolo G, Cosmi L, Romagnani S, Annunziato F (2006). Regenerative and immunomodulatory potential of mesenchymal stem cells. Curr Opin Pharmacol.

[24] D’Ippolito G, Diabira S, Howard GA, Roos BA, Schiller PC (2006). Low oxygen tension inhibits osteogenic differentiation and enhances stemness of human MIAMI cells. Bone.

[25] Grayson WL, Zhao F, Bunnell B, Ma T (2007). Hypoxia enhances proliferation and tissue formation of human mesenchymal stem cells. Biochem Biophys Res Commun.

[26] Lennon DP, Edmison JM, Caplan AI (2001). Cultivation of rat marrow-derived mesenchymal stem cells in reduced oxygen tension: effects on in vitro and in vivo osteochondrogenesis. J Cell Physiol.

[27] Ren H, Cao Y, Zhao Q, Li J, Zhou C, Liao L (2006). Proliferation and differentiation of bone marrow stromal cells under hypoxic conditions. Biochem Biophys Res Commun.

[28] Jaiswal N, Haynesworth SE, Caplan AI, Bruder SP (1997). Osteogenic differentiation of purified, culture-expanded human mesenchymal stem cells in vitro. J Cell Biochem.

[29] Allen JW, Bhatia SN (2003). Formation of steady-state oxygen gradients in vitro: application to liver zonation. Biotechnol Bioeng.

[30] Das R, Jahr H, van Osch GJ, Farrell E (2010). The role of hypoxia in bone marrow-derived mesenchymal stem cells: considerations for regenerative medicine approaches. Tissue Eng Part B Rev.

[31] Potier E, Ferreira E, Andriamanalijaona R, Pujol JP, Oudina K, Logeart-Avramoglou D (2007). Hypoxia affects mesenchymal stromal cell osteogenic differentiation and angiogenic factor expression. Bone.

[32] Wang JA, Chen TL, Jiang J, Shi H, Gui C, Luo RH (2008). Hypoxic preconditioning attenuates hypoxia/reoxygenation-induced apoptosis in mesenchymal stem cells. Acta Pharmacol Sin.

[33] Ohnishi S, Yasuda T, Kitamura S, Nagaya N (2007). Effect of hypoxia on gene expression of bone marrow-derived mesenchymal stem cells and mononuclear cells. Stem Cells.

[34] Hung SC, Pochampally RR, Hsu SC, Sanchez C, Chen SC, Spees J (2007). Short-term exposure of multipotent stromal cells to low oxygen increases their expression of CX3CR1 and CXCR4 and their engraftment in vivo. PLoS One.

[35] Wang Y, Deng Y, Zhou GQ (2008). SDF-1alpha/CXCR4-mediated migration of systemically transplanted bone marrow stromal cells towards ischemic brain lesion in a rat model. Brain Res.

[36] Rosova I, Dao M, Capoccia B, Link D, Nolta JA (2008). Hypoxic preconditioning results in increased motility and improved therapeutic potential of human mesenchymal stem cells. Stem Cells.

[37] Bordone L, Guarente L (2005). Calorie restriction, SIRT1 and metabolism: understanding longevity. Nat Rev Mol Cell Biol.

[38] Salminen A, Kaarniranta K (2009). SIRT1: regulation of longevity via autophagy. Cell Signal.

[39] Michalopoulos GK (2007). Liver regeneration. J Cell Physiol.

[40] Garcia-Rodriguez JL, Barbier-Torres L, Fernandez-Alvarez S, Gutierrez-de Juan V, Monte MJ, Halilbasic E (2014). SIRT1 controls liver regeneration by regulating bile acid metabolism through farnesoid X receptor and mammalian target of rapamycin signaling. Hepatology.

[41] Jin J, Iakova P, Jiang Y, Medrano EE, Timchenko NA (2011). The reduction of SIRT1 in livers of old mice leads to impaired body homeostasis and to inhibition of liver proliferation. Hepatology.

[42] Wang Y, Jiang Y, Fan X, Tan H, Zeng H, Wang Y (2015). Hepato-protective effect of resveratrol against acetaminophen-induced liver injury is associated with inhibition of CYP-mediated bioactivation and regulation of SIRT1-p53 signaling pathways. Toxicol Lett.

[43] Bellet MM, Masri S, Astarita G, Sassone-Corsi P, Della Fazia MA, Servillo G (2016). Histone deacetylase SIRT1 controls proliferation, circadian rhythm, and lipid metabolism during liver regeneration in mice. J Biol Chem.

[44] Fujiyoshi M, Ozaki M (2011). Molecular mechanisms of liver regeneration and protection for treatment of liver dysfunction and diseases. J Hepatobiliary Pancreat Sci.

[45] Chen R, Dioum EM, Hogg RT, Gerard RD, Garcia JA (2011). Hypoxia increases sirtuin 1 expression in a hypoxia-inducible factor-dependent manner. J Biol Chem.

[46] Pillai VB, Sundaresan NR, Gupta MP (2014). Regulation of Akt signaling by sirtuins: its implication in cardiac hypertrophy and aging. Circ Res.

[47] Overturf K, al-Dhalimy M, Ou CN, Finegold M, Grompe M (1997). Serial transplantation reveals the stem-cell-like regenerative potential of adult mouse hepatocytes. Am J Pathol.

