# Supplementary figures and images for: Determination of optimized oxygen partial pressure to maximize the liver regenerative potential of the secretome obtained from adipose-derived stem cells
Source: Stem Cell Res Ther. 2017 Aug 3;8:181. doi: 10.1186/s13287-017-0635-x (PMC5543744; doi:10.1186/s13287-017-0635-x)

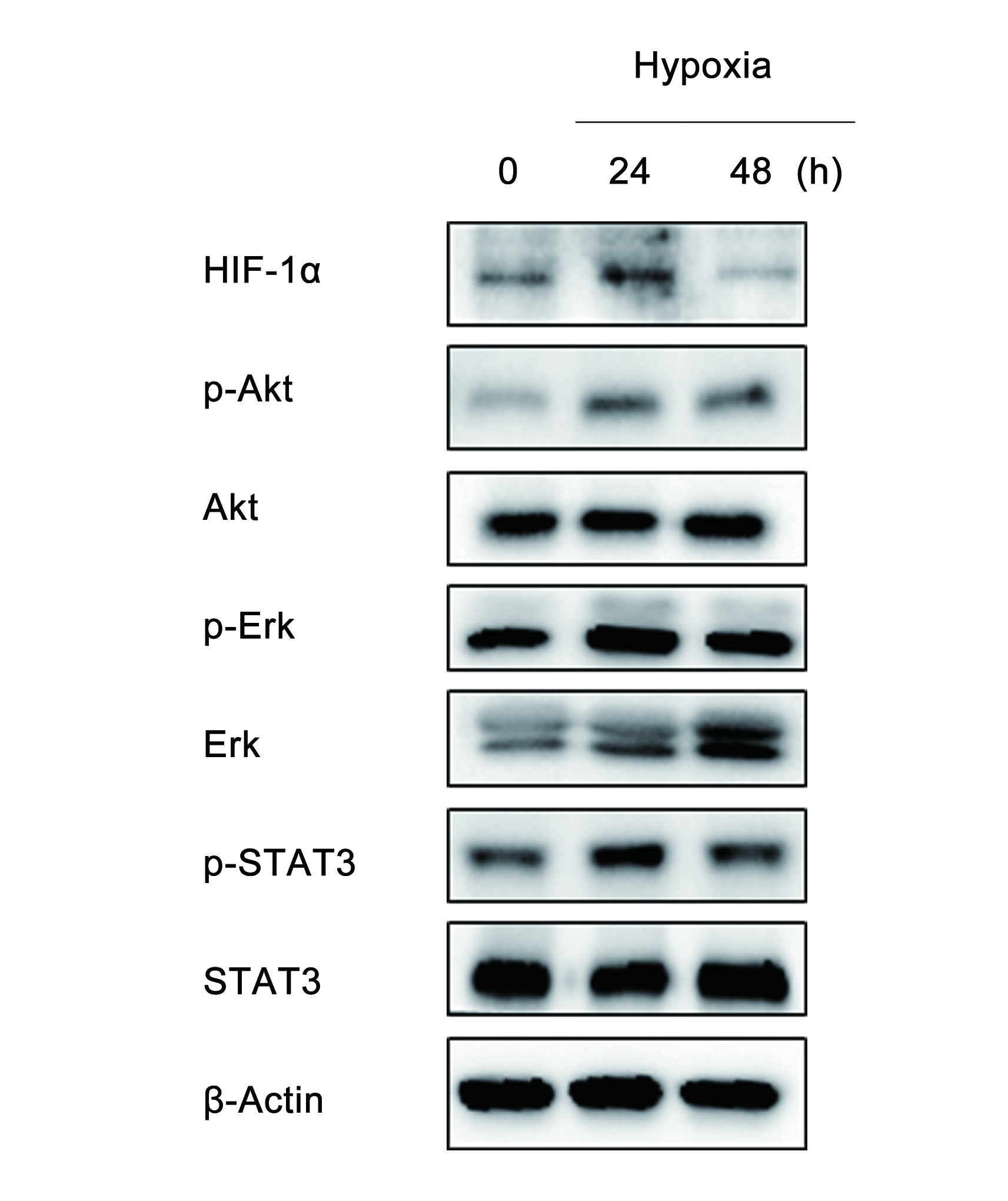

Supplement: Supplementary file 1 — Western blot analysis showing the comparison of the expression of various signaling mediates between 24-h and 48-h culturing periods. Expression of HIF-1α, p-Akt, p-STAT3, and p-ERK was further increased after 24-h incubation, leading us to decide 24-h hypoxic culturing of stem cells. (TIF 1429 kb) [file 13287_2017_635_MOESM1_ESM.tif]

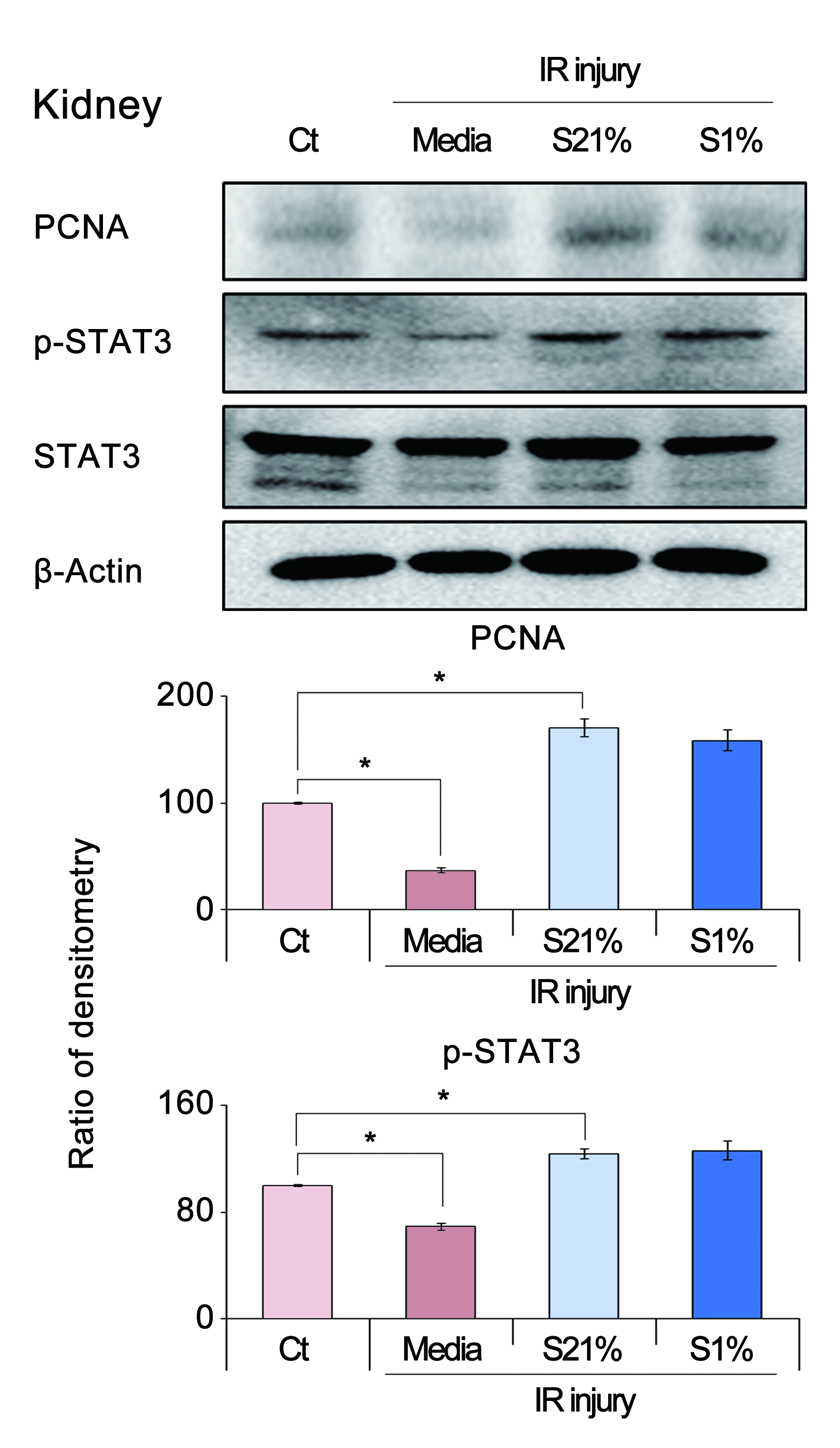

Supplement: Supplementary file 2 — Western blot analysis showing the effects of the secretome with 21% and 1% pO2 on the IR-induced human renal cells (HK2 cells), respectively. There was no significant difference in the expression of these markers between the two different pO2 secretome groups. (TIF 1830 kb) [file 13287_2017_635_MOESM2_ESM.tif]
